# Activation of oxytocin neurons in the paraventricular nucleus drives cardiac sympathetic nerve activation following myocardial infarction in rats

**DOI:** 10.1038/s42003-018-0169-5

**Published:** 2018-10-04

**Authors:** Ranjan K. Roy, Rachael A. Augustine, Colin H. Brown, Daryl O. Schwenke

**Affiliations:** 1 Department of Physiology–HeartOtago, University of Otago, Dunedin, 9054 New Zealand; 2 Brain Health Research Centre, University of Otago, Dunedin, 9054 New Zealand; 3 Centre for Neuroendocrinology, University of Otago, Dunedin, 9054 New Zealand

## Abstract

Myocardial infarction (MI) initiates an increase in cardiac sympathetic nerve activity (SNA) that facilitates potentially fatal arrhythmias. The mechanism(s) underpinning sympathetic activation remain unclear. Some neuronal populations within the hypothalamic paraventricular nucleus (PVN) have been implicated in SNA. This study elucidated the role of the PVN in triggering cardiac SNA following MI (left anterior descending coronary artery ligation). By means of c-Fos, oxytocin, and vasopressin immunohistochemistry accompanied by retrograde tracing we showed that MI activates parvocellular oxytocin neurons projecting to the rostral ventral lateral medulla. Central inhibition of oxytocin receptors using atosiban (4.5 µg in 5 µl, i.c.v.), or retosiban (3 mg/kg, i.v.), prevented the MI-induced increase in SNA and reduced the incidence of ventricular arrhythmias and mortality. In conclusion, pre-autonomic oxytocin neurons can drive the increase in cardiac SNA following MI and peripheral administration of an oxytocin receptor blocker could be a plausible therapeutic strategy to improve outcomes for MI patients.

## Abstract (web summary)

Roy et al. showed that activation of parvocellular pre-autonomic oxytocin neurons increased sympathetic nerve activity following myocardial infarction. This and other aberrant physiological changes induced by acute myocardial infarction were decreased by oxytocin receptor antagonists, hinting to their potential therapeutic role.

## Introduction

An acute myocardial infarction (MI) is associated with severe damage to the myocardium that impairs cardiac function. This damage is exacerbated by sustained over-stimulation of the nerves that control heart function, specifically sympathetic nerve activity (SNA)^1^. The initial increase in cardiac SNA within the first hours following MI is known to contribute, at least in part, to the generation of ventricular arrhythmias^1,2^, which is ultimately responsible for sudden heart failure and death^3^. Once established, this sympathetic hyper-excitation is essentially irreversible and facilitates permanent structural and functional damage of the heart^4^.

Despite the advent of coronary reperfusion therapy and other advances in the clinical treatment of myocardial infarction, early arrhythmias facilitated by an increased SNA still often prove fatal^5^. Preventing sympathetic activation has therefore emerged as a promising target for the development of new therapeutic options because it targets the origin of the increase in SNA before it can contribute to sudden heart failure^6^.

To date, the pathological mechanisms underpinning sympathetic activation following MI remain to be fully elucidated, although peripheral neural reflexes and central integrative pathways have been implicated^7^. Sympathetic traffic emanating primarily from the rostral ventral lateral medulla (rVLM) is shaped and refined by input from the nucleus tractus solitarius (NTS), which is the site where peripheral afferent signals converge. Although the rVLM and NTS have classically been considered the principal central nervous system (CNS) nuclei that modulate sympathetic outflow^8,9^, activation of the hypothalamic paraventricular nucleus (PVN) has now emerged as a key regulator in the sustained elevation in SNA, at least in chronic heart failure^10–13^.

The PVN consists of a heterogeneous group of magnocellular and parvocellular neurons that are largely clustered into anatomically distinct divisions^14^. Magnocellular neurons, which project to the posterior pituitary, serve an endocrine function through the secretion of oxytocin or vasopressin directly into the circulation^15^. Within the parvocellular division, a distinct sub-population of pre-autonomic neurons (some of which also release oxytocin or vasopressin) project to the rVLM^16–18^ and have been implicated in the modulation of SNA^17,19^.

To the best of our knowledge, no study has identified the neuronal pathways within the CNS that are activated to increase cardiac SNA immediately following MI. Yet, it is this early period, before the increase in SNA has become irreversibly established, where pharmacological intervention has the greatest opportunity of blocking or reversing sympathetic activation^20^ to improve patient outcome.

The main aim of this study was to identify whether pre-autonomic PVN neurons drive the early increase in cardiac SNA following acute MI. We used neuronal retrograde tracing and immunohistochemistry to show that acute MI selectively activates a sub-population of parvocellular pre-autonomic oxytocin neurons that project to the rVLM. These results are likely to be of clinical significance since the administration of an oxytocin receptor blocker, whether it be centrally (intracerebroventricular (i.c.v.)) or even peripherally (intravenous (i.v.)), effectively prevents the central-mediated increase in SNA following MI.

## Results

### MI increases c-Fos expression in the parvocellular PVN

**Fig. 1:**
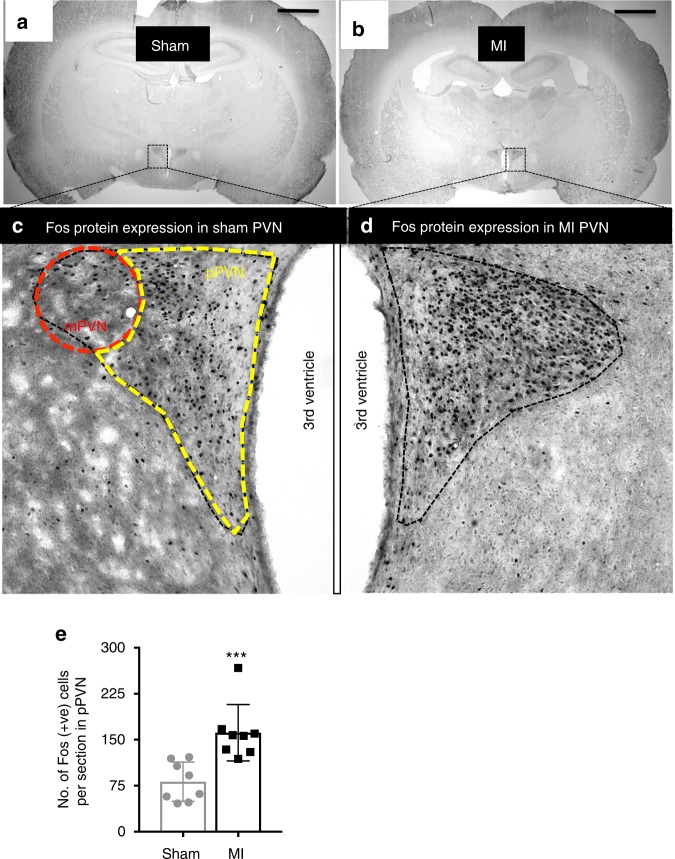
Parvocellular and magnocellular divisions of the PVN. Photomicrographs of coronal sections through the brain at the level of the paraventricular nucleus (PVN) following sham (**a**) or acute myocardial infarction (MI; **b**). **c**, **d** High magnification of the PVN, illustrating the division of the parvocellular (yellow) and magnocellular (ref) regions. Black staining indicates c-Fos-positive nuclei. **e** A bar graph quantifying the number of c-Fos-positive cells in the parvocellular division of the PVN for sham (*n* = 8) and MI rats (*n* = 8). All data are presented as mean ± SEM. ***P < 0.001 vs sham, unpaired *t*-test. Scale bars = 50 μm

Immunohistochemical staining of coronal brain sections for c-Fos, as a marker of neuronal activation, showed that acute MI triggered widespread neuronal activation throughout the PVN, with little c-Fos protein expression in other brain areas at the level of the PVN (Fig. 1). In particular, the number of c-Fos-positive neurons within the parvocellular PVN (pPVN) of MI rats was almost double that of sham rats (Fig. 1c).

### MI increases oxytocin, not vasopressin neuronal activation

**Fig. 2:**
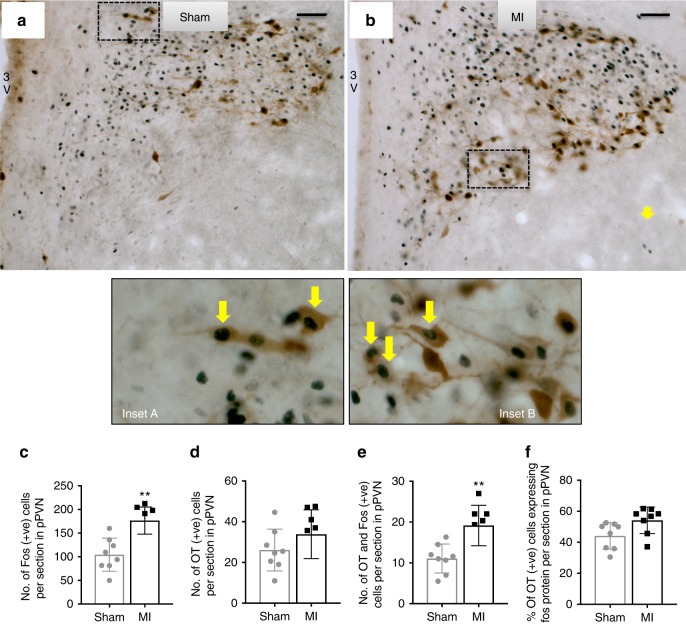
Parvocellular PVN oxytocin neuronal activation following acute MI. Representative photomicrographs of coronal sections of the PVN following **a** sham or **b** acute myocardial infarction (MI). Black staining indicates c-Fos-positive nuclei, brown staining indicates oxytocin-positive cytoplasm, and black surrounded by brown staining indicates co-localization of c-Fos protein with oxytocin (yellow arrows). Insets **a**, **b** High-power magnification of the dashed-boxes taken from the low magnification micrographs. The bar graphs (mean ± SEM) show the number of: **c** c-Fos-positive cells, **d** oxytocin-positive cells, **e** cells co-localized with oxytocin+c-Fos-protein, and **f** percentage of oxytocin-positive cells expressing c-Fos protein per section in bilateral parvocellular division of the PVN (pPVN). **P *<* 0.01 signficant difference between MI rats (*n* = 8) and sham rats (*n* = 8; unpaired *t*-test). Scale bars = 50 μm. 3V third ventricle, pPVN parvocellular paraventricular nucleus

Parvocellular PVN neurons predominantly express oxytocin or vasopressin^21,22^. Therefore, we used double label immunohistochemistry to determine which of these two phenotypes was primarily activated following acute MI. In this series of experiments, we again observed a higher number of c-Fos-positive neurons in the pPVN of MI rats compared to sham rats (Fig. 2a–c). Although there appeared to be a higher number of oxytocin-positive neurons in the pPVN of MI rats compared to sham rats, it was not significant (Fig. 2d). Moreover, while the number of oxytocin-positive neurons that co-expressed c-Fos protein was higher in the pPVN of MI rats compared to that of sham rats (Fig. 2e), the proportion of oxytocin-positive neurons co-expressing c-Fos was similar for MI rats and sham rats (Fig. 2f).

**Fig. 3:**
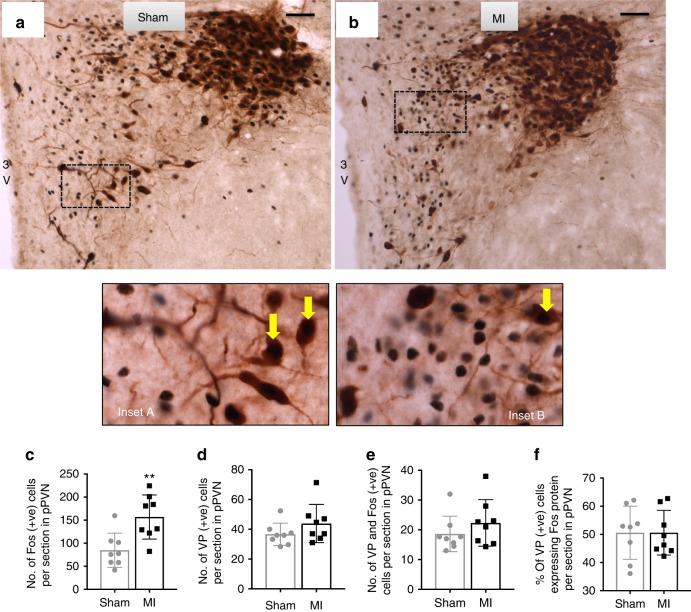
Parvocellular PVN vasopressin neuronal activation following acute MI. Representative photomicrographs of coronal sections through the brain at the level of the PVN following **a** sham or **b** acute myocardial infarction (MI). Black staining indicates c-Fos-positive nuclei, brown staining indicates vasopressin-positive cytoplasm, and black nuclei surrounded by brown staining indicates co-localization of c-Fos protein with vasopressin (yellow arrows). Insets **a**, **b** High-power magnification of the dashed-boxes taken from the low magnification micrographs. The bar graphs (mean ± SEM) show the number of: **c** c-Fos-positive cells, **d** vasopressin-positive cells, **e** cells co-localized with vasopressin+c-Fos-protein, and **f** percentage of oxytocin-positive cells expressing c-Fos protein per section in bilateral parvocellular division of the PVN. **P *<* 0.01 significant difference between MI rats (*n* = 8) and sham rats (*n* = 8; unpaired *t*-test). Scale bars = 50 μm. 3V third ventricle, pPVN parvocellular paraventricular nucleus

In contrast to oxytocin-positive neurons, the number of pPVN vasopressin-positive neurons was not different between MI and sham rats (Fig. 3a–d). Similarly, there was no difference in the number (Fig. 3e) or the proportion (Fig. 3f) of vasopressin-positive neurons that co-expressed c-Fos.

### Activated parvocellular PVN neurons project to the rVLM

**Fig. 4:**
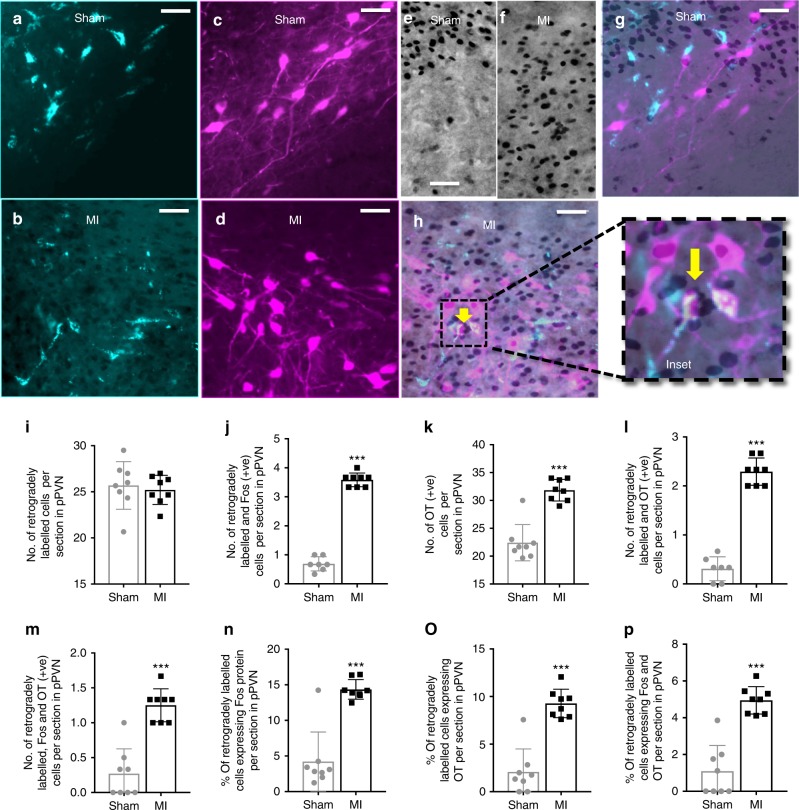
Retrograde labeling of rostral ventrolateral medulla-projecting pPVN oxytocin neurons. Representative photomicrographs of coronal sections through the brain at the level of the PVN showing: **a**, **b** fluorescently stained retrogradely labeled cells and **c**, **d** fluorescently stained oxytocin-positive cells, **e**, **f** Bright-field c-Fos-positive cells and **g**, **h** the co-localization of retrograde label, oxytocin, and c-Fos-protein (yellow arrow) for MI rats (*n* = 8) and sham rats (*n* = 8). Inset: high-power magnification showing a close-up of neurons co-labeled for c-Fos + OT + retrograde tracer. Bar graphs (mean ± SEM) present the number of **I** retrogradely labeled cells, **j** retrogradely labeled + c-Fos-positive cells, **k** oxytocin-positive cells, **l** retrogradely labeled + oxytocin-positive cells, **m** retrogradely labeled + c-Fos-positive + oxytocin-positive cells, as well as the proportion (%) of **n** retrogradely labeled cells expressing c-Fos protein, **o** retrogradely labeled cells expressing oxytocin, and **p** retrogradely labeled cells expressing c-Fos and oxytocin, per section in the left parvocellular division of the PVN. ***P *<* 0.001 significant difference between MI rats (*n* = 8) and sham rats (*n* = 8; unpaired *t*-test). Scale bars = 25 μm. pPVN parvocellular paraventricular nucleus

**Fig. 5:**
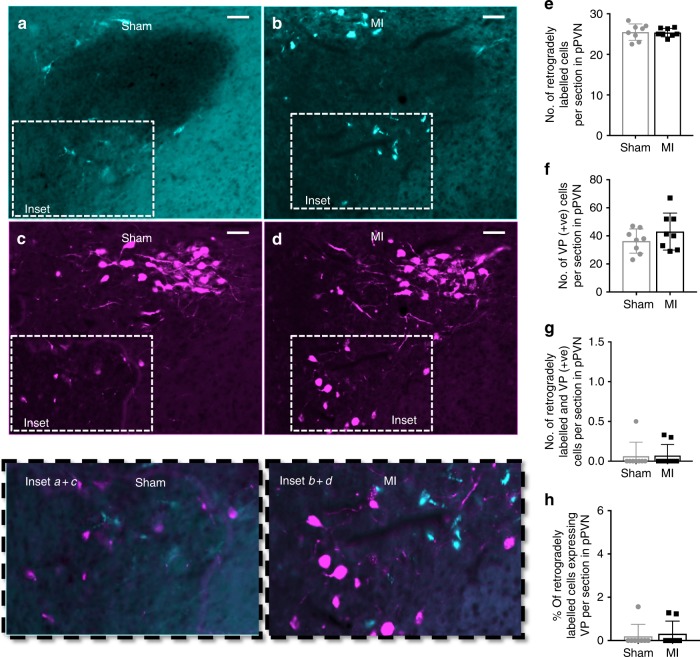
Retrograde labeling of rostral ventrolateral medulla-projecting pPVN vasopressin neurons. Representative photomicrographs of coronal sections through the brain at the level of the PVN showing: **a**, **b** retrogradely labeled cells (fluorescent green), **c**, **d** vasopressin-positive cells (fluorescent red;) and (inset **a **+** b**; inset **c **+** d**) the co-localization of retrograde label and vasopressin protein. The bar graphs (mean ± SEM) present the number of **e** retrogradely labeled cells, **f** vasopressin-positive cells, **g** the number of retrogradely labeled + vasopressin-positive cells, as well as **h** percentage of retrogradely labeled cells expressing vasopressin, per section in the left parvocellular division of the PVN. There was no difference in the number of fluorescently stained cells (red and green) in MI rats (*n* = 8) compared to sham rats (*n* = 8; unpaired *t*-test). Scale bars = 50 μm. pPVN parvocellular paraventricular nucleus

Having identified that acute MI selectively activates pPVN oxytocin neurons, we next determined whether these neurons project to the rVLM by injection of retrograde tracer into the rVLM (Supplementary Figure. 1) and subsequent co-staining pPVN neurons for c-Fos and either oxytocin (Fig. 4a–h) or vasopressin (Fig. 5a–d). A key result from this study is that the number and proportion of retrogradely labeled pPVN oxytocin-positive neurons co-expressing c-Fos was higher in MI rats compared to sham rats (Fig. 4i–p). In contrast to oxytocin neurons, there were virtually no pPVN vasopressin neurons retrogradely labeled from the rVLM (Fig. 5g), which was similar for both MI and sham rats (Fig. 5g, h). Hence, it appeared that acute MI selectively activated rVLM-projecting oxytocin neurons in the pPVN.

### Oxytocin receptor blockade prevents sympathetic activation

Considering acute MI activates a distinct sub-population of pPVN pre-autonomic oxytocin neurons, we tested whether central oxytocin receptor antagonism could prevent sympathetic activation following acute MI. Moreover, to determine whether oxytocin receptor blockade could be a clinically translatable option for the treatment of MI, we assessed the efficacy of peripherally administered Retosiban, an oxytocin receptor blocker that can cross the blood–brain barrier, for preventing sympathetic activation.

### Acute MI-induced arrhythmogenesis

**Fig. 6:**
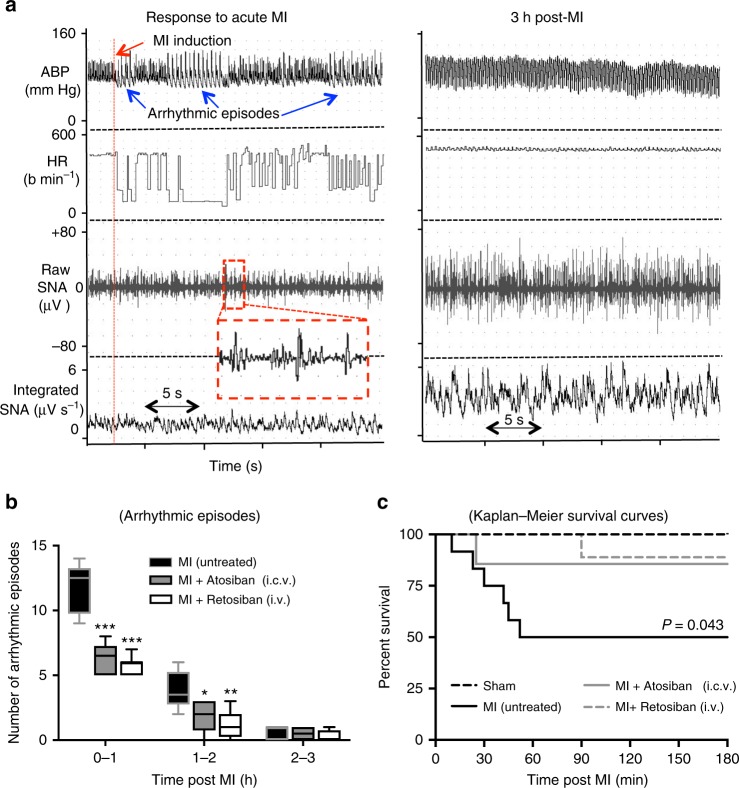
Effect of atosiban (i.c.v.) and retosiban (i.v.) on arrhythmia and mortality following acute MI. **a** A typical ‘Chart’ recording showing an example of arrhythmic episodes within the first minute following LAD coronary occlusion (i.e., myocardial infarction (MI)), and the subsequent increase in cardiac SNA at 180 min post MI. The inset (red box) shows a close-up view of several impulse profiles. **b** The incidence of arrhythmic episodes (mean ± SEM) each hour for consecutive 3 h following MI in untreated MI rats (Untreated; *n* = 6), or rats treated with atosiban (MI + atosiban, 4.5 µg in 5 µl i.c.v. *n* = 6) or retosiban (MI + retosiban, 3 mg/kg, i.v. *n* = 8). Both atosiban and retosiban significantly reduced the incidence of arrhythmic episodes following acute MI, compared to saline-treated MI rats (*P *<* 0.05, **P *<* 0.01, ***P *<* 0.001, unpaired *t*-test). **c** Kaplan–Meier survival analysis showing a greater mortality rate in MI + saline rats (*n* = 12) compared to MI + retosiban rats (*n* = 9; *P* = 0.043) and MI + atosiban rats (*n* = 7; NS) within 3 h of the MI. None of the sham rats (*n* = 6) died during the experiment. SNA sympathetic nerve activity

Cardiac arrhythmias were evident within the first minute of left anterior descending (LAD) coronary artery occlusion in all MI rats and persisted for at least 2 h post MI (Fig. 6a), although the incidence of arrhythmic episodes subsequently decreased with time (Fig. 6b). Importantly, the incidence of arrhythmias was lower in the MI rats that received an oxytocin receptor antagonist either centrally (atosiban, 4.5 µg in 5 µl, i.c.v.) or intravenously (retosiban, 3 mg/kg) (Fig. 6b).

### Survival

Six of 12 MI rats treated with saline died (50% mortality) from sudden heart failure triggered by ventricular arrhythmias within 3 h following MI (Fig. 6c). Both atosiban treatment (i.c.v.) and retosiban treatment (i.v.) reduced mortality following acute MI (15 and 11% mortality, respectively; *P* = 0.04 for retosiban). No sham rats died during experimentation.

### Cardiac sympathetic nerve activity

**Fig. 7:**
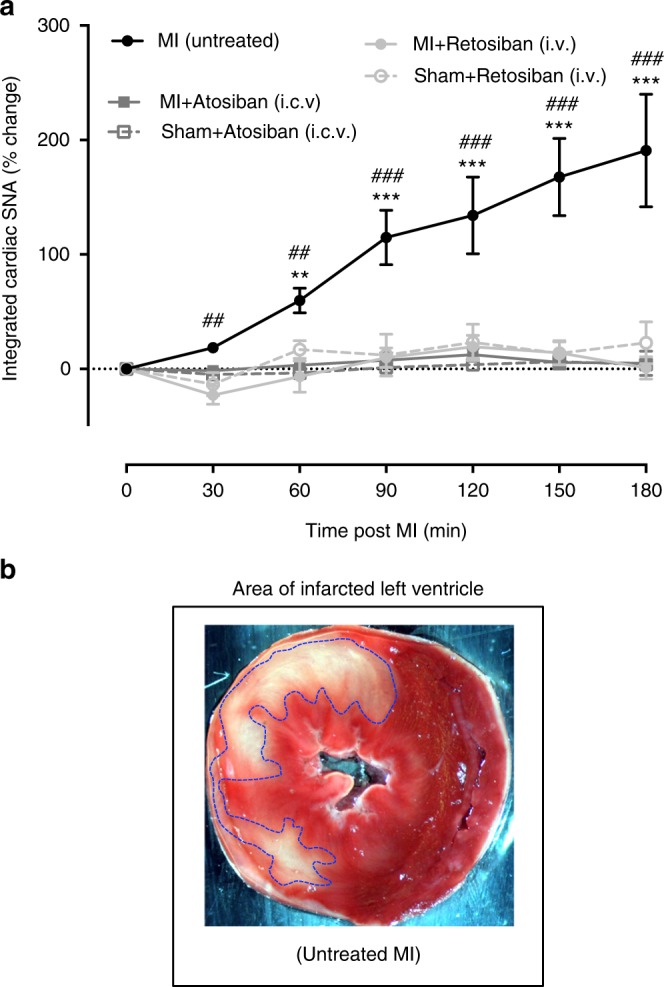
Effect of atosiban (i.c.v.) and retosiban (i.v.) on cardiac sympathetic nerve activity following acute MI. **a** Changes in cardiac sympathetic nerve activity (% increase in cardiac SNA – of integrated area of the raw nerve signal) in SHAM rats treated with atosiban (4.5 µg in 5 µl i.c.v. *n* = 6) or retosiban (3 mg/kg, i.v. *n* = 6), or untreated MI rats (*n* = 6), or M -rats treated with atosiban (i.c.v. *n* = 6) or retosiban (3 mg kg^−1^, i.v. *n* = 8). There was a significant main effect of TIME (*F*_(6, 90)_ = 10.96, *P* *<* 0.0001, two-way RM ANOVA), TREATMENT (*F*_(2, 15)_ = 12.37, *P* *=* 0.0007, two-way RM ANOVA), and a significant TIME × TREATMENT interaction (*F*_(12, 90)_ = 5.69, *P* *<* 0.0001, two-way RM ANOVA). *P *<* 0.05, ***P *<* 0.001 vs pre-MI (time zero), ^#^*P* *<* 0.05, ^##^#*P* *<* 0.001 vs MI + retosiban and vs MI + atosiban, Bonferroni’s post hoc test. All data are presented as mean ± SEM. **b** Representative transverse section of a heart slice stained with tetrazolium chloride (TTC) as a quantitative means of assessing of infarct size. The viable myocardium absorbs the TTC stain and forms a reddish pink pigment. In contrast, the infarcted myocardium remains unstained pale-white (encircled by blue dashed line)

In sham rats treated with either atosiban (i.c.v.) or retosiban (i.v.), cardiac SNA remained stable for the 3 h recording period. In MI rats treated with saline, cardiac SNA progressively increased (190 ± 49%) over 3 h following the infarct (Fig. 7a). In contrast, the administration of either atosiban or retosiban immediately after the MI completely prevented any subsequent MI-induced increase in cardiac SNA (Fig. 7a).

### Infarct size

The size of the infarct of the left ventricular wall (Fig. 7b) was similar between untreated MI rats given saline (47 ± 2%) and MI rats given atosiban (41 ± 2%) or retosiban (43 ± 3%).

### Arterial blood pressure and heart rate

There was no significant change in arterial blood pressure (ABP) or estimated heart rate (eHR) over 3 h post MI (or sham) in any of the groups (Supplementary Figure 2).

## Discussion

Despite advances in the treatment of acute heart failure in recent decades, effective therapeutic strategies for averting or reversing sympathetic activation following acute MI have remained largely elusive because, at least in part, the pathological mechanisms responsible for increasing cardiac SNA following acute MI are yet to be fully elucidated. This study demonstrates that acute MI activates a distinct population of pPVN oxytocin neurons that project to the rVLM. Importantly, intravenous administration of the oxytocin receptor antagonist, retosiban, completely prevented the MI-induced increase in cardiac SNA, which likely contributed to the reduced incidence of ventricular arrhythmias and improve survival. Given that retosiban crosses the blood–brain barrier^23^, and that selective central blockade of oxytocin receptors (atosiban) prevents sympathetic activation, it appears likely that retosiban acts centrally to prevent oxytocin neuronal activation of the rVLM. Regardless of its site of action, retosiban has potential as a novel therapy for the acute treatment of MI.

While the role of hypothalamic nuclei is well characterized for sustaining the increase in SNA in animal models of chronic heart failure^24–26^, one distinct finding of this study is that we have identified the pPVN as a crucial center for triggering the increase in cardiac SNA in the very early stages (within 90 min) following acute MI. We quantified neuronal activation using c-Fos immunohistochemistry. One limitation, however, is that c-Fos protein is not fully expressed until ~90 min after the initial stimulus^27,28^, meaning we could not pinpoint the exact moment that the oxytocin neurons were activated. In spite of this limitation, one key advantage of using c-Fos immunohistochemistry is that, since the c-Fos is expressed only within the cell nuclei, it can be used in combination with tract-tracing procedures to identify the phenotypic variation of c-Fos-labeled neurons^29^. Accordingly, c-Fos protein has been extensively used for over three decades as a reliable neuronal marker to identify key brain regions involved with autonomic regulation^10,27,30–33^.

The parvocellular division of the PVN comprises neurons that project either to the median eminence^19^, where they serve a neuroendocrine role to control anterior pituitary function, or to other areas of the brain including the spinal cord and rVLM, where they play a pre-autonomic role^16^. Moreover, pPVN pre-autonomic neurons comprise multiple different neuronal phenotypes^21^, of which oxytocin neurons that project to the rVLM account for ~3% of the population^34^. In agreement with previous reports, we also identified that oxytocin neurons accounted for 1–2% of all retrogradely labeled parvocellular neurons, at least in sham animals. Remarkably, we noted that the proportion of neurons in which oxytocin protein could be detected using immunohistochemistry increased sixfold within 90 min of an MI. This ability to rapidly increase the number of oxytocin-expressing neurons is likely due to ~80% of the parvocellular pre-autonomic neurons already expressing the oxytocin gene^21^, although under basal conditions, transcription of the oxytocin gene in the majority of neurons is presumably below the threshold to translate sufficient oxytocin protein to be detected by immunohistochemistry. Thus, many of the previously undetectable oxytocin pPVN neurons were already primed to facilitate an increase in oxytocin protein synthesis following acute MI.

Remarkably, the increase in the number of pPVN oxytocin neurons appeared to be selective for pre-autonomic neurons projecting to the rVLM, rather than a ubiquitous increase throughout the pPVN. These results perhaps reflect a physiological adaptation for accommodating the increased neuronal traffic to the rVLM following acute MI. Importantly, the mechanism by which an MI triggers the intracellular pathways associated with neuronal plasticity is an area of research that warrants further investigation.

To date, emerging evidence implicates pPVN oxytocin neurons as potential modulators of SNA^35,36^. Indeed, the direct microinjection of oxytocin into the rVLM appears to facilitate a sympathetic-mediated increase in mean arterial pressure and heart rate^37^. Hence, in this study it is reasonable to surmise that an ~30% increase in pPVN pre-autonomic oxytocin neuronal activation is sufficient to elicit a 190% increase in cardiac SNA following acute MI, as reported in this study.

As mentioned above, there are some pPVN neurons that project to the spinal cord^14,17,38^ of which a sub-population is oxytocinergic^39^. Hence, we cannot rule out the possibility that some of these oxytocinergic projections to the spinal cord could be driving cardiac SNA post MI. Interestingly, studies have reported that, at least in chronic heart failure, there is a preferential increase in neuronal activity of pPVN neurons projecting to the rVLM, while pPVN neurons that project to the spinal cord remain largely unchanged^17^. Whether this is also true in the very early stages following acute MI remains unknown.

In contrast to sympathetic modulation, hypothalamic oxytocin neurons have been implicated in the modulation of parasympathetic nerve activity, at least in chronic heart failure, facilitating a functional and structural cardioprotective effect^40^. The role that these cardiac vagal neurons play in modulating cardiac function in the very early stages following MI remains an intriguing area of future research.

Patients who experience an acute MI are commonly prescribed a range of therapeutic interventions such as β-adrenergic receptor blockers, fibrinolytics, anti-platelet drugs, angiotensin-converting enzyme inhibitors, and analgesics as part of standard clinical practice^41,42^. Yet, considering the negative impact that sympathetic activation has on patient outcome^43^, it is somewhat perplexing that an effective sympathoinhibitory therapy has not yet emerged as part of current clinical practice^42,44^. Unfortunately, even coronary reperfusion therapy is limited in reversing or averting the early increase in cardiac SNA^5^.

Considering that pPVN pre-autonomic oxytocin neurons are activated following acute MI, we subsequently used direct electrophysiological recordings of cardiac SNA in vivo to assess whether oxytocin receptor blockade, using retosiban, was effective at preventing sympathetic activation following acute MI. The advantages of using retosiban, compared to other oxytocin receptor antagonists are that; (i) as a non-peptide antagonist, retosiban can cross the blood–brain barrier so that an intravenous injection will access the brain^23^, which is clinically important for the immediate treatment for MI patients; (ii) retosiban is >18,000-fold more selective for oxytocin receptors than vasopressin (V1a) receptors^45^; (iii) therapeutic doses of retosiban are well tolerated with no adverse side effects on cardiac function^46^; and (iv) the half-life of 1.4 h^47^ means that a single injection should remain effective until the patients can be hospitalized. Currently, retosiban has entered phase III clinical trials for treatment of preterm labor, after the successful outcome of a phase II pilot dose-ranging study^47^.

Perhaps one of the most pertinent observations from this study is that retosiban prevented sympathetic activation following acute MI, reduced arrhythmic incidence, and improved survival. Although the mechanisms that trigger arrhythmias are largely dictated inherently by the damage incurred by the injured myocardium, such as acidosis, hypoxia, and Ca^2+^ and K^+^ ionic imbalances, an adverse increase in SNA is recognized as a key extrinsic driver for arrhythmogenesis following acute MI^5,48,49^, as evident in our study.

Since retosiban was administered intravenously, it was unclear as to whether retosiban was acting peripherally or centrally to suppress sympathetic activation. However, the observations that select central inhibition of oxytocin receptors (atosiban, i.c.v.) produced similar sympathoinhibitory effects as that seen when retosiban was administered peripherally supports the idea that retosiban is centrally suppressing SNA. Further studies are now essential to confirm the exact site, within the CNS, at which oxytocin receptor blocker is effective at suppressing SNA. Indeed, it is possible that retosiban’s sympathoinhibitory effects are mediated through oxytocin signaling pathways other than the pPVN, as proposed in this study. Regardless of the mechanism or site of action, retosiban’s potent sympathoinhibitory effects, its ability to cross the blood–brain barrier, and its exceptional safety profile, make it a strong candidate for a phase I clinical trial as an early treatment for MI patients.

In conclusion, we have used immunohistochemistry and retrograde labeling to identify a unique sub-population of pPVN pre-autonomic oxytocin neurons that project to the rVLM and that are selectively activated in the very early stages following an acute MI. In vivo electrophysiological recordings of cardiac SNA reveals the critical importance of this sub-population of oxytocin neurons for mediating the increase in cardiac SNA and, in doing so, identifies the highly specific oxytocin receptor antagonist, retosiban, as a promising therapy for the first-line treatment of patients who suffer an acute MI.

## Methods

### Animals

Experiments were conducted on male Sprague Dawley rats (10 weeks old; body weight ~250–350 g). All rats were on a 12 h light/dark cycle and provided with food and water *a*d libitum. All experiments were approved by the Animal Ethics Committee of the University of Otago, New Zealand, and conducted in accordance with the New Zealand Animal Welfare Act, 1999, and associated guidelines. The data sets generated during and/or analyzed during the current study are available from the corresponding author on reasonable request.

### Induction of MI

Rats were deeply anesthetized with an intraperitoneal injection of pentobarbital sodium (60 mg kg^−1^; Sigma-Aldrich, St. Louis, MO, USA) for immunohistochemistry experiments or urethane (1.5 g kg^−1^; Sigma-Aldrich) for electrophysiology experiments. Adequate anesthesia was confirmed by elimination of the limb withdrawal reflex. Throughout the experiment body temperature was maintained at 38 °C using a rectal thermistor coupled with a thermostatically controlled heating pad (BWT- 100, Bio Research Center Co., Ltd.)^49^. The trachea was cannulated and the lungs ventilated using a Harvard rodent ventilator (model 680). The inspirate gas was enriched with O_2_ (~50% O_2_) and the ventilator settings were adjusted (tidal volume ~ 3.5 ml; breathing rate ~80 min^−1^) to maintain arterial PCO_2_ normocapnic. The femoral artery and vein were cannulated for measurement of systemic ABP and fluid administration (saline at 3 ml h^−1^), respectively^5^. A left thoracotomy was performed between the second and third ribs to expose the anterior surface of the left ventricle. A 7.0 Prolene suture was loosely placed around the LAD coronary artery which was located between the appendage of the left atrium and the base of the pulmonary artery^5^.

### Immunohistochemistry for c-Fos, oxytocin, and vasopressin

Following 90 min of LAD occlusion (MI) or sham operation, rats were transcardially perfused with 4% paraformaldehyde (PFA) in phosphate buffer solution (0.2 M, pH 7.6). Brains were removed and post-fixed in 4% PFA overnight. Brains were then immersed in 30% sucrose and subsequently sectioned using a freezing microtome at −21 °C (Lyca, SM2400, Germany) into 30 μm coronal sections through the PVN (−1.56 mm to −1.80 mm relative to Bregma)^50^.

To immunostain for c-Fos protein, sections were washed thoroughly and incubated in tris-buffered saline (TBS) solution containing hydrogen peroxide (H_2_O_2_) (0.9%) and methanol (40%) for 15 min to prevent endogenous peroxidase activity. Sections were then incubated in rabbit anti-c-Fos antibody (SC-52, Santa Cruz Biotechnology, Santa Cruz, CA, USA 1:2500) for 48 h at 4 °C, followed by incubation in biotinylated goat anti-rabbit IgG antibody (Vector, BA-1000; 1:200) for 90 min at room temperature. Sections were incubated in Vectastain Elite ABC Solution (Vector Laboratories, Burlingame, USA) for 90 min at room temperature, followed by incubation in diaminobenzidine (DAB) substrate kit solution with nickel enhancement (Vector Lab, Burlingame, USA) to visualize c-Fos. Sections were examined at regular intervals under a bright-field microscope. When a visible black precipitate was observed against the background, the sections were washed in TBS to stop the reaction.

Double label immunohistochemistry for c-Fos protein with either oxytocin or vasopressin was also performed using chromagen labeling with DAB without nickel enhancement. The c-Fos protein was first labelled as described earlier. Subsequently, the procedure was repeated to label either the oxytocin or vasopressin antigen, although, instead of using a biotinylated secondary antibody for the oxytocin labeling, a peroxidase-labeled secondary antibody was used. To specifically label for oxytocin protein, mouse monoclonal anti-oxytocin antibody (MAB5296, Millipore, 1:20000) and horseradish peroxidase horse anti-mouse IgG antibody (Vector Laboratories, PI-2000; 1:200) were used. To label for vasopressin protein, guinea pig monoclonal anti-arginine vasopressin antibody (Peninsula Laboratories, San Carlos, CA; 1:25,000) and biotinylated goat anti-guinea pig secondary antibody were used (Vector, BA7000; 1:200). Negative controls were run with omission of primary antibodies and showed no non-specific staining.

Immunostained sections were mounted onto gelatin-coated slides and left to dry at room temperature. Slides were cover slipped with a mounting media containing distyrene, plasticiser, and xylene (DPX) (Sigma-Aldrich, USA) and left overnight to dry in a fume hood.

### Retrograde tracing from the rVLM

To characterize neuronal projections from the parvocellular PVN to the rVLM, fluorescent immunohistochemistry for oxytocin or vasopressin was performed in brain sections from rats that were injected with retrograde tracer into the rVLM. Standard aseptic surgical procedures were used for the injection of green fluorescent microsphere beads (Lumafluor Inc., Durham, USA), that emit fluorescence at 460 nm^51^, directly into the rVLM 1 week prior to MI induction.

Rats were anesthetized with 1–5% isoflurane (1 l min^−1^ of O_2_) placed in a small animal stereotaxic frame. The tip of a flame-pulled microinjection needle was positioned in the rVLM based on the coordinates of Paxinos and Watson (12 mm posterior to the bregma, 2.1 mm lateral to the midline, and 10 mm ventral to the skull surface)^50^ and 100 nl of green fluorescent microspheres was injected over 5 min using a Nanoject II micromanipulator device (Drummond Scientific Company, cat. no. 3-000-205A, Philadelphia, USA). Only the brains from those rats that showed evidence of correct tracer positioning completely within the rVLM (Supplementary Figure. 1) were used for subsequent immunohistological staining for c-fos and oxytocin.

Following recovery and post-operative care, rats were returned to their standard housing conditions where they remained and were monitored for 1 week. On day 7 post surgery, rats were subjected to the MI or sham protocol and their brains removed and sectioned, as described above.

The protocol for fluorescent immunohistochemistry was similar to that described for DAB immunohistochemistry with the exception that (1) endogenous aldehyde activity was blocked by incubating the tissue sections in sodium borohydride (NaBH_4_, 0.1%) for 20 min at the beginning of the immunohistochemistry protocol, and (2) the secondary antibodies used to label oxytocin and vasopressin were fluorescent-tagged; Alexa Fluor 568 goat anti-mouse (A 11031, Molecular Probs, Oregon, USA; 1:500) and Alexa Fluor 568 goat anti-guinea pig (Life Technologies, USA; 1:500), respectively. The sections were then stained for c-Fos protein using chromagen labeling with DAB as previously described. Following immunostaining, tissues were mounted, dried, and cover slipped with Vectashield mounting medium (Vector Laboratories Inc., Burlingame, CA, USA).

### Immunohistochemistry data analysis

#### DAB immunohistochemistry data analysis

The c-Fos-positive, oxytocin-positive, and vasopressin-positive cell bodies were counted manually using Olympus AX51 bright-field microscope, with the experimenter blinded to the experimental groups. The cell bodies were counted on both sides of the pPVN in three sections and the mean number of labeled cells was calculated for each rat. Where double labeling was completed, the number of neurons with oxytocin or vasopressin that co-localized with c-Fos protein was also counted.

#### Fluorescent immunohistochemistry data analysis

Oxytocin-positive and vasopressin-positive cell bodies were counted using Olympus AX51 epifluorescent microscope with FITC (for retrograde label) and Texas Red filters (for oxytocin and vasopressin) with the experimenter blinded to the experimental groups. Retrograde tracer injection into the RVLM predominantly labels the ipsilateral side of the PVN^16,18^ and so quantification was carried out only on the ipsilateral side of the pPVN to the retrograde tracer injection site. The number of cells with either retrograde-label, oxytocin-positive, or vasopressin-positive cells were counted, as well as the number of cells that co-expressed retrograde label and either oxytocin or vasopressin.

### Electrophysiological recording of cardiac sympathetic nerve activity

Using urethane-anesthetized rats, a left thoracotomy was performed between the first and second ribs to expose and isolate the stellate ganglion. The cardiac sympathetic nerve was identified as a branch from the stellate ganglion, dissected free of surrounding connective tissue, sectioned, and the proximal section (containing efferent fibers) was placed on a pair of platinum recording electrodes^5,49^. The signal was filtered (low-cutoff 0.1 kHz; high-cutoff 1 kHz) and amplified^5^ (BMA-200, AC/DC Bioamplifier, USA) and subsequently passed through an amplitude discriminator (model WD-2, Dagan Corp., MN, USA) for counting nerve discharge frequency (impulse frequency). The femoral artery was cannulated for the continuous measurement of ABP. The interval between the arterial systolic peaks was used as an estimation of heart rate (eHR).

One group of rats received an i.c.v. injection of atosiban. Using a stereotaxic frame, the tip of a 27-gauge stainless steel cannula was positioned in the right lateral cerebral ventricle based on the coordinates of Paxinos and Watson (0.8 mm posterior to the bregma, 1.5 mm lateral to the midline, and 5.0 mm ventral to the skull surface). The distal end of the cannula was connected to a 10 µl Hamilton syringe for subsequent drug administration. Correct positioning of the i.c.v. catheter was confirmed after each experiment by staining with Evans blue dye (5 µl)^49^.

Cardiac SNA, ABP, and eHR were continuously recorded for 20 min prior to occlusion of the LAD coronary artery, and for consecutive 3 h after LAD occlusion (MI), followed by an injection within 5 min of the infarct of either (a) saline i.v. (untreated MI, *n* = 6), (b) i.c.v.: atosiban (4.5 µg in 5 µl; Sigma CAS Number 90779-69-4; MI + atosiban; *n* = 6), or (c) i.v.: retosiban (3 mg kg^−1^; GSK 221-149 A, USA; MI + retosiban; *n* = 8). Sham animals did not undergo LAD occlusion, but did receive (a) an i.c.v. injection of atosiban (Sham + atosiban, *n* = 6) or (b) an i.v. injection of retosiban (Sham + retosiban, *n* = 6).

### Electrophysiology data analysis

Raw cardiac SNA and ABP were continuously sampled at 4 kHz and 400 Hz, respectively, using a PowerLab data-acquisition system (model 8/S, AD Instruments Ltd, New Zealand). The raw nerve signal was rectified and integrated (0.5 s resetting interval) online, and the integrated nerve signal was displayed in real time. The scale of variability in SNA within groups was reduced by omitting the highly-variable background ‘noise’ levels (i.e., zero nerve activity) from the recorded electroneurogram^5^.

At the end of each experiment, the electroneurogram was continuously recorded as the rat was killed by an intra-cardiac injection of 1 M KCl, which elicited a maximum increase in cardiac SNA within the first 10 s of the heart stopping^5^. Subsequently, after the animal died, only background noise contributed to the overall recorded electroneurogram. During post-experiment analysis, this ‘noise’ was subtracted from the pre-recorded SNA. Moreover, we were able to verify that the maximal increase in SNA in response to KCl (>250 µV s^−1^) was markedly more than that in response to MI (~50–80 µV s^−1^ increase) or between groups of rats and, thus, a 'ceiling' effect was unlikely to confound the SNA results of this study. To further avoid potential variability between groups, SNA data were normalized by assessing the magnitude of response (% increase) to each manipulation^5^.

### Measurement of infarct size

At the completion of each electrophysiology experiment, the rat was killed and the heart excised and sectioned into 2 mm horizontal slices down the vertical plane. The sections were then stained with 2,3,5-triphenyltetrazolium chloride (TTC) (Sigma-Aldrich, Inc., MO, USA) and subsequently fixed in 10% formalin for 20 min. Slices were mounted and photographed. Total infarct size was determined by measuring the area of the infarction for each slice, multiplying the area by the slice thickness, and summing the area of all slices. Infarct size was presented as a percentage of the total left ventricular wall^49^.

### Statistical analysis

Statistical analyses were performed using Prism (v6.0; GraphPad Software Inc.). All results are presented as mean ± SEM. Immunohistochemical experiments were analyzed using unpaired *t*-tests. In electrophysiology experiments, two-way analysis of variance (ANOVA; repeated measures) was used to test significance for (i) temporal changes in recorded variables following LAD occlusion and (ii) differences between MI + saline and MI + retosiban (i.v) or MI + atosiban (i.c.v) groups; where the *F*-ratio was significant, Bonferroni’s post hoc tests were completed. The Kaplan–Meier survival analysis was performed to compare survival curves between the different groups of MI rats. A *P* value ≤ 0.05 was predetermined as the level of significance for all statistical analyses.

## Electronic supplementary material

Supplementary Figures

## Data Availability

The data that support the findings of this study are available within the article and supplementary files, or available from the corresponding authors upon request.
