# Supplementary material for: Activation of oxytocin neurons in the paraventricular nucleus drives cardiac sympathetic nerve activation following myocardial infarction in rats
Source: Commun Biol. 2018 Oct 4;1:160. doi: 10.1038/s42003-018-0169-5 (PMC6172223; doi:10.1038/s42003-018-0169-5)
Supplement: Supplementary file 1 — Supplementary Figures [file 42003_2018_169_MOESM1_ESM.pdf]

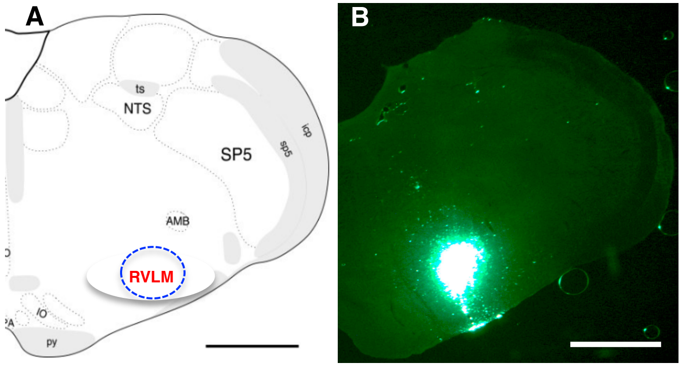

Supplementary Figure 1. Verification of site for retrograde tracer injection.
(A) Schematic diagrams modified from (Paxinos & Watson 2006), illustrating the anatomical position for retrograde tracer injection. Retrograde tracer was aimed to the rVLM, indicated by the blue dashed circle. (B) Represents a 'hit' based on the observation that retrograde tracer encompassed the entire RVLM. NTS; nucleus tractus solitarius, AMB; ambiguus nucleus, SP5; Spinal tegmental tract.

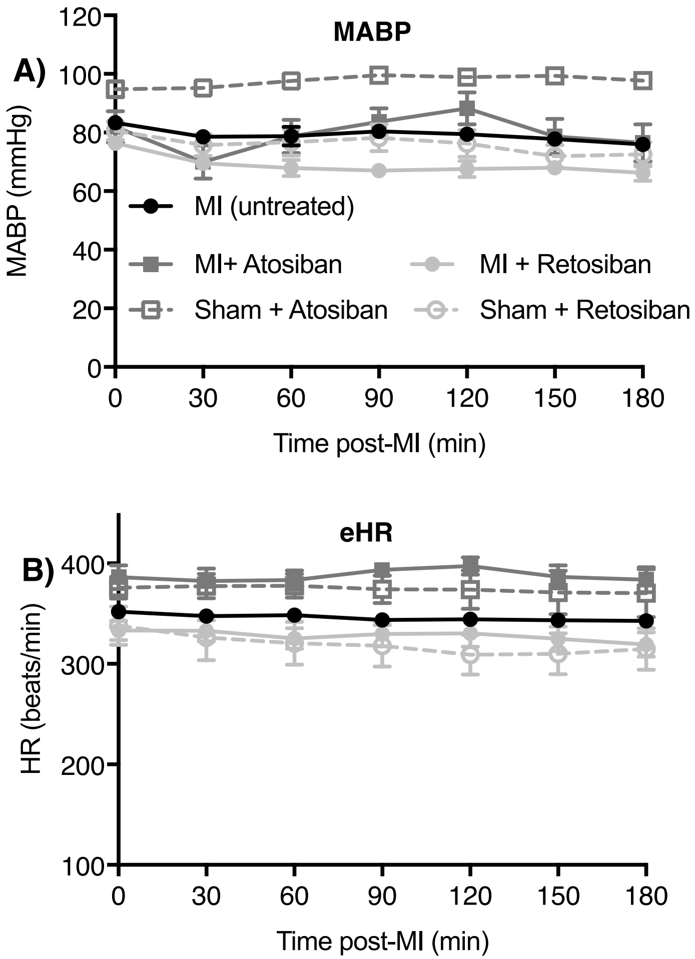

Supplementary Figure 2. Effect of atosiban (i.c.v.) and retosiban (i.v.) on arterial blood pressure and estimated heart rate following acute MI.

(A) and (B) Graphs showing mean arterial blood pressure (ABP) and estimated Heart rate (HR), respectively, in SHAM rats treated with atosiban (4.5 µg in 5 µl i.c.v. n = 6) or retosiban (3 mg/kg, i.v. n = 6, or untreated MI-rats (n = 6), or MI-rats treated with atosiban, (i.c.v. n = 6) or retosiban (3 mg/kg, i.v. n = 8). (A) There was no significant main effect of TIME ($F (6, 84) = 2.68$, $P = 0.673$, two-way RM ANOVA) or TREATMENT ($F (2, 15) = 2.49$, $P = 0.117$, two-way RM ANOVA) and no TIME x TREATMENT interaction ($F (12, 90) = 0.78$, $P = 0.674$, two-way RM ANOVA). All data are presented as mean ± SEM. (B) There was no significant main effect of TIME ($F(6,90) = 12.63$, $P < 0.079$, two way RM ANOVA), TREATMENT ($F(2,12) = 14.69$, $P = 0.672$, two-way RM ANOVA) and no TIME x TREATMENT interaction ($F (12, 84) = 1.81$, $P = 0.106$, two-way RM ANOVA).
